# Delineating the source of resistance to bean common mosaic virus (BCMV) and bean common mosaic necrosis virus (BCMNV) in common bean (*Phaseolus vulgaris*) cultivars of Jammu and Kashmir, a North-Western Himalayan region

**DOI:** 10.3389/fmicb.2025.1614122

**Published:** 2025-06-24

**Authors:** Dasari Meghanath, Sumiah Wani, Sabiya Bashir, Shahjahan Rashid, Andleeb Javaid, Zahoor Ahmad Dar, Shabir Hussain Wani, Parvaze A. Sofi, Gowhar Ali, Aflaq Hamid

**Affiliations:** ^1^DNA Fingerprinting and Advanced Plant Virology Laboratory, AICRP-NSP, Sher-e-Kashmir University of Agricultural Sciences and Technology of Kashmir, Srinagar, India; ^2^Dryland Agricultural Research Station (DARS), Sher-e-Kashmir University of Agricultural Sciences and Technology of Kashmir, Budgam, India; ^3^Division of Genetics and Plant Breeding, Faculty of Agriculture (FoA), Sher-e-Kashmir University of Agricultural Sciences and Technology of Kashmir, Sopore, India

**Keywords:** BCMV, BCMNV, resistance, *I*-gene, *bc-3* gene

## Abstract

Bean common mosaic virus (BCMV) and bean common mosaic necrosis virus (BCMNV) are among the most challenging constraints for common bean production in Northern states of India due to their easy transmission through aphids and seeds. Highly valuable Indian common bean varieties and landraces are more susceptible to BCMV and BCMNV and very few varieties exhibit resistance to these viruses. Resistance towards these viruses is governed by a single dominant (*I*) gene and a few recessive genes (*bc-1, bc-2, bc-3, bc-4, bc-u^d^*, and *bc-u^r^*). This study aims to identify common bean genotypes bearing multiple resistant genes, each working with a different mode of action. A total of 123 genotypes of common beans were mechanically inoculated with BCMV and BCMNV isolates and molecular markers (SW13, ROC11, BCMV-CAPS, ENM-FWe/Rve) were used to identify the presence of two major resistant genes (*I* and *bc-3*). Out of these, 23 genotypes were found phenotypically resistant to both viruses. Furthermore, molecular screening was performed in which 13 hypersensitive resistant genotypes bearing a single dominant gene (*I*) were confirmed through SW13 and BCMV-CAPS markers. Additionally, ROC11/420, ENMF/R markers identified 4 genotypes bearing the recessive (*bc-3*) gene conferring complete resistance to the virus without executing hypersensitive response (HR). A valuable gene combination of both *I, bc-3* (*Ibc-3*, Host group-12) genes in 3 genotypes was also established in the screened germplasm. However, in 3 phenotypically resistant genotypes, neither the *I* gene nor *bc-3* gene was identified. The virus accumulation in the resistant genotypes was also understood properly through a time course experiment in a qPCR assay. This extensive identification of resistant common bean genotypes against BCMV and BCMNV can be readily included in the common bean breeding program of the Northern states of India for virus resistance.

## 1 Introduction

Bean common mosaic virus (BCMV) and bean common mosaic necrosis virus (BCMNV) are one of the most widespread viruses infecting common beans (*Phaseolus vulgaris* L.) and other cultivated legumes with worldwide distribution (Morales and Bos, 1988; Drijfhout and Morales, 2005; Morales, 2006). These viruses belong to the genus *Potyvirus* within the family *Potyviridae*. They possess a single-stranded (+) RNA genome of approximately 10 kilobases that encodes a large polyprotein precursor which is subsequently cleaved into 10 functional proteins (Adams et al., 2005; Olspert et al., 2015). Both viruses are non-persistently transmitted by probing aphids and are seed-borne, with a transmission efficiency of up to 80% (Wani et al., 2023; Morales, 2006). The major symptoms induced by BCMV on bean plants were mosaic, necrosis, chlorosis, etiolation and deformation, whereas BCMNV strains induce severe necrosis of bean plants (Drijfhout, 1978; Hamid et al., 2014; Rashid et al., 2022; Wani et al., 2023).

In India, especially in the Northern Himalayan region, valuable local bean varieties are becoming increasingly susceptible to diverse strains of BCMV and BCMNV, leading to heavy yield losses in hilly areas compared to the Northern plains. Also, BCMNV-induced whole plant necrosis was reported from Jammu and Kashmir (Hamid et al., 2014; Rashid et al., 2022) in which identifying resistant sources goes unrecognized. To overcome these viruses, we must identify and utilize resistant genotypes through germplasm screening and incorporate them into breeding programs. Resistance to BCMV and BCMNV is conferred by a single dominant (*I*) gene and six recessive genes namely *bc-1, bc-2, bc-3, bc-4, bc-u^d^*, and *bc-u^r^* (Ali, 1950; Soler-Garzón et al., 2024; Soler-Garzón et al., 2021a; Soler-Garzón et al., 2021b). The dominant (*I*) gene confers resistance against BCMV-BCMNV through a hypersensitive response (HR) on the primary leaves, whereas recessive genes confer absolute resistance toward specific viral strains (Drijfhout, 1978). Genotypes exhibiting HR present nervure localized necrosis on the primary inoculated leaves and demonstrate the presence of the dominant “*I*” gene for resistance (Drijfhout, 1978; Ogliari and Castano, 1992; Vallejos et al., 2006; Feng et al., 2015). At temperatures exceeding 30°C, some bean cultivars possessing the *I* gene experience strong whole plant necrosis (WPN), when infected with BCMNV and BCMV: NL-2 and NL-6 strains (Cadle-Davidson and Jahn, 2005). This interaction leads to a disease known as black root (Collmer et al., 2000; Feng et al., 2014). Hence, to prevent the WPN induced by BCMNV and BCMV (NL-2 and NL-6), genotypes bearing the “*I*” gene must be protected with extra “*bc*” recessive genes, especially in high-temperature areas (Collmer et al., 2000; Cadle-Davidson and Jahn, 2005; Feng et al., 2017). Although the non-necrotic strains cannot induce WPN in genotypes bearing the dominant *I* gene, the presence of an extra recessive gene will protect the bean plants from extreme HR when the temperature exceeds 30°C and provide additional protection (Ogliari and Castano, 1992).

Recessive resistance to BCMV-BCMNV is conferred by six recessive genes: four strain-specific *bc-1, bc-2, bc-3*, and *bc-4* genes and two strain-unspecific *bc-u^r^* and *bc-u^d^* resistant genes (Soler-Garzón et al., 2021a; Soler-Garzón et al., 2021b; Soler-Garzón et al., 2024; Nisa et al., 2024). Strain-specific genes confer resistance to specific strains of BCMV-BCMNV. However, the *bc-3* gene is exceptional in providing resistance to common beans against all strains of BCMV-BCMNV (Drijfhout, 1978; Miklas et al., 1998) except for a BCMV isolate 1755a (PG-VIII) that was able to overcome the *bc-3* resistance (Feng et al., 2015). The strain-non-specific gene (*bc-u*^d^*)* is required for the complete action of the strain-specific genes (Drijfhout, 1978; Soler-Garzón et al., 2021a; Soler-Garzón et al., 2021b). A new gene “*bc-u^r^*” at the *Bc-u* locus on chromosome Pv05 was recently recognized as another strain-non-specific gene that interacts with *bc-u^d^* and also with the other recessive genes for conferring resistance against BCMV (Nisa et al., 2024; Soler-Garzón et al., 2024). To identify common bean varieties possessing these multiple resistance genes, reliable methods are necessary. Marker-assisted selection can be used as a consequence to efficiently select genotypes with desirable resistance genes. Molecular markers have been identified to be effective in identifying resistance genes in common beans against BCMV and BCMNV. Haley et al. (1994) identified a Randomly Amplified Polymorphic Marker (RAPD): OW13, linked to the *I* gene, which was later developed into a Sequence Characterized Amplified Region (SCAR) marker: SW13 (Melotto et al., 1996) that was more reliable and widely used. Molecular markers were also developed for the identification of recessive genes (Tang and Feng, 2022). Since most of these markers were dominant, progeny testing is necessary to distinguish between homozygous and heterozygous plants. Therefore, the use of co-dominant Cleaved Amplified Polymorphic Sequence (CAPS) marker was suggested for precise and efficient selection of BCMV-resistant genotypes compared to dominant markers (Bello et al., 2014).

As a proof of concept, the current study was conducted to identify common bean genotypes bearing the dominant *I* gene, recessive *bc-3* gene and combination of both *I* and *bc-3* genes (*Ibc-3)* for resistance with different modes of action based on phenotypic evaluation and molecular screening utilizing SCAR and CAPS markers. The pathogenicity and resistance mechanism during virus-plant interaction were studied. The resistant genes were later cloned, sequenced and aligned to identify the nucleotide differences and their implementation against virus resistance.

## 2 Materials and methods

### 2.1 Collection and maintenance of plant materials, viral isolates and inoculation method

123 genotypes of common beans, including 53 collections obtained from the National Bureau of Plant Genetic Resources (NBPGR)-New Delhi and 68 lines maintained by All India Co-ordinated Research Project (AICRP)- Seed Crops, AICRP-Pulses and Division of Genetics and Plant Breeding (Wadura, SKUAST-K) with truly unknown disease reaction to BCMV-BCMNV were evaluated in this study. All plants were grown under two treatments (T1: BCMV, T2: BCMNV) and three replicates in a growth chamber under ambient conditions of 26°C temperature and 70% relative humidity. For every genotype, non-inoculated control and mock-inoculated plants were maintained. The BCMV and BCMNV isolates (MW675689; OK094708), identified in our previous study (Rashid et al., 2022), were maintained through periodical propagation on *Nicotiana benthamiana* plants and were used to mechanically inoculate test plants at the primary leaf stage. The viral inoculum was prepared by homogenizing infected leaf tissue in 100 mM potassium phosphate buffer (pH 7.0) with 0.5% celite added directly to the viral inoculum just before inoculation (Wani et al., 2025). Ten days after planting, the first trifoliate leaves were mechanically inoculated with both BCMV and BCMNV isolates individually to identify the presence of the dominant hypersensitive *I* gene and recessive *bc-3* gene. All the treatments were performed in triplicates with non-inoculated control and mock-inoculated plants maintained separately.

### 2.2 Screening of common bean germplasm against BCMV-BCMNV

#### 2.2.1 Phenotypic evaluation

Disease reaction and symptoms of each virus were recorded every 2 days post inoculation (dpi) from 0 to 30 dpi and the genotype is classified as resistant/susceptible at 30th dpi. Disease severity was scored on a 0–3 scale according to Odu (Odu et al., 2004), where 0 = no disease symptoms/HR on plants, 1 = mild foliar disease symptoms, 2 = moderate foliar disease symptoms and 3 = severe distortion, malformation of leaves or stem and stunting. Resistant genotypes that exhibited no symptoms or HR were re-sown and re-inoculated to confirm resistance. Both symptomatic and asymptomatic plants were further tested using an RT-PCR assay to confirm the presence of the virus.

#### 2.2.2 Genotypic evaluation

123 genotypes were screened for *I* and *bc-3* gene resistance irrespective of the plant reaction to the virus (Table 2). Two SCAR markers (SW13 and ROC11) and two CAPS markers (BCMV-CAPS and ENM-FWe/Rve) were used for selection and their thermal conditions are given in Table 1. These markers were chosen due to their proven reliability and close linkage to the *I* and *bc-3* genes. SCAR markers were initially used to identify the genotypes bearing resistant genes and CAPS markers were later used for revalidation. Pooled leaves of three replicates per genotype were collected at 30 dpi and stored in a deep freezer at −80°C. DNA was extracted from the young leaves of 10-day-old plants using a DNeasy plant mini kit (Qiagen). Total RNA was isolated from 100 mg of leaf tissue using TRIzol reagent as per the user guidelines (Invitrogen, Thermo Scientific). 1 μg of RNA was used as a template for cDNA synthesis using the Revert-Aid cDNA synthesis kit (Thermo Scientific) in a total volume of 20 μL. 2 μL of cDNA was used in the RT-PCR assay to test the presence of virus in both susceptible and resistant plants. PCR cycling conditions are listed in Table 1. PCR reactions were performed in 25 μL volumes, each containing 1 μL of 50 ng genomic DNA as a template, 12.5 μL of GoTaq green dye master mix (Promega, Madison, United States), 10 μM of each primer (20 pmol for SW13). For CAPS markers, 5 μL of PCR products were digested with 1 μL of 10X Reaction buffer (Thermo Scientific) and 1 μL of the restriction enzyme (*Taq*1 for *I* gene, *Rsa*1 for *bc-3* gene) in a total volume of 15 μL. The digested products were then separated on a 2% agarose gel. The complete nucleotide sequence of both *I* and *bc-3* genes corresponding to the BCMV-CAPS and ENM-FWe/Rve amplified product were purified and sanger sequenced at Medauxin genomics, Bangalore, http://www.medauxin.com/. The sequences were aligned and analyzed using the BioEdit 7.0 sequence alignment editor. BLASTn analysis of the ENM-FWe/Rve amplicon sequence was performed to identify similarities with known *bc-3* gene sequences in reported cultivars.

**TABLE 1:** List of Markers used in the study and their thermal conditions.

Marker	Gene	Sequence and thermal conditions	Amplicon length	References
SW13	*I*	5′-CACAGCGACATTAATTTTCCTTTC-3′ 5′-CACAGCGACAGGAGGAGCTTATTA-3′ One cycle at 94°C for 4 min; 35 cycles of 94°C-10 s, 63°C- 40 s, 72°C- 2 min.	690 bp	Haley et al., 1994
CAPS (*Taq1*)		5′-AGGAGGAAGAACGGTGGTC-3′ 5′-TTTGGTGGTAATTTGAAAATGG-3′ One cycle at 94°C for 5 min; 35 cycles of 94°C-30 s, 58°C- 30 s, 72°C- 30 s. followed by restriction digestion	300 bp	Bello et al., 2014
ROC11	*bc-3*	5′-CCAATTCTCTTTCACTTGTAACC-3′ 5′-GCATGTTCCAGCAAACC-3′ One cycle at 94°C for 2 min; 30 cycles at 92°C-10 s, 65°C- 10 s, 72°C for 25 s.	420 bp	Johnson et al., 1997
ENM-FWe/Rve (*Rsa1*)		5′- ACCGATGAGCAAAACCCTA -3′ 5′- CAACCAACTGGTATCGGATT-3′ One cycle at 95°C-3 min; 40 cycles at 94°C-20 s, 58°C-20 s, 72°C – 20 s. Followed by restriction digestion	541 bp (381/160)	Naderpour et al., 2010
BCMV CP (For RT-PCR and qPCR)	Coat protein	5′- AGTTGTTCCTCGGCATTCAAA-3′ 5′- TACGGCCTCTCGGAATTTCT-3′ One cycle at 95°C-2 min; 35 cycles 95°C-30 s, 55°C-30 s, 72°C-1 min. Final extension of 72°C for 10 min.	414 bp	Wani et al., 2025
BCMNV (for RT-PCR and qPCR)		5′- ATGAACAGTGTGGCGAAGTG-3′ 5′- GCTTTGTTGGGCTCTTCAAC-3′ One cycle at 95°C-2 min; 35 cycles 95°C-30 s, 55°C-30 s, 72°C-1 min. Final extension of 72°C for 10 min.	834 bp	Rashid et al., 2022

### 2.3 qRT-PCR analysis

qPCR assay was carried out to study the best resistant genotypes inhibiting the replication and systemic movement of the virus in different resistant plants (*I, bc-3*, and *Ibc-3*). For assays, resistant genotypes possessing the *I* (WB-352), *bc-3* (EC-127645), and *Ibc-3* (EC-116117) genes for resistance were selected along with a highly susceptible plant (IC-437141). All plants were inoculated with BCMV-BCMNV isolates and RNA was extracted from these genotypes at two development stages (Day 4 and Day 8). For every genotype, uninoculated resistant plants were used as an untreated control. Reactions were performed in the Rotor-Gene Q Real-Time PCR system (QIAGEN) using a 20 μL reaction mixture, including 10 μL SYBR green master mix (Thermo Scientific), 1 μL each of forward and reverse primers (BCMV-CP and BCMNV, 10 μm, Table 1), 2 μL cDNA and 6 μL of Nuclease-free water. The cycling conditions were set at an initial hold of 95°C for 2 min, followed by cycling for 35 times at 95°C-30 s, 60°C-30 s and 72°C- 60 s. Following the final PCR cycle, melting curve analysis was performed on the samples by heating them from 70 to 95°C, with a rise of 0.3°C for each step for the detection of specific and non-specific PCR products. Each sample was assayed in three replicates, including the actin gene, used as an internal control. The comparative quantification report was used to construct the box plots using REST 2008 software (QIAGEN). For both qPCR and RT-PCR, BCMV and BCMNV specific primers (Rashid et al., 2022; Wani et al., 2025) were used to detect both viruses (Table 1).

## 3 Results

### 3.1 Plant reaction to BCMV/BCMNV

Based on the phenotypic evaluation from the screening experiment, 23 genotypes were found resistant to both BCMV and BCMNV. The resistant plants were categorized into two groups, hypersensitive and absolute, based on the presence or absence of necrosis symptoms on the inoculated leaves. The results were presented under three experimental groups based on the selection of resistant genotypes assisted by molecular markers: Group 1: characterized by the sole presence of the *I* gene, Group 2: characterized by the sole presence of the *bc-3* gene, and Group 3: characterized by the presence of both *I* and *bc-3* genes (*Ibc-3*).

### 3.2 Phenotypic and genotypic evaluation of plant materials bearing *I* gene

**FIGURE 1:**
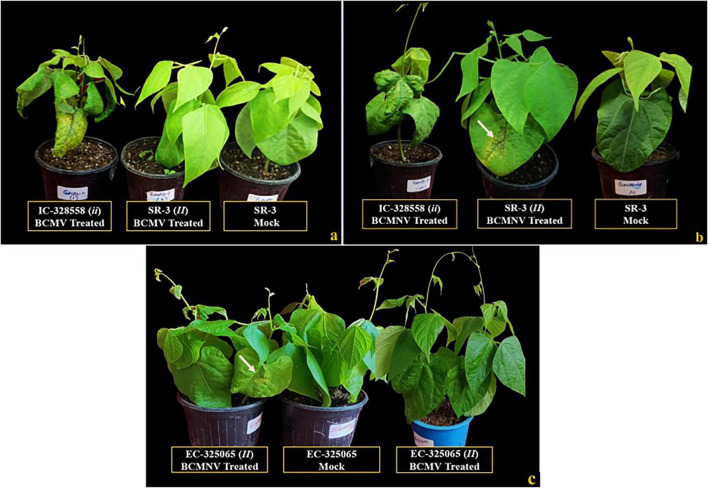
Resistant genotypes identified for the presence of the Dominant “*I*” gene. **(a)** All treatments inoculated with BCMV and a mock (SR-3, conditioning resistance to BCMV without HR). **(b)** All treatments inoculated with BCMNV and a mock (SR-3, conditioning resistance through an HR to BCMNV). **(c)** EC-325065 inoculated with BCMNV conditioning resistance through HR and without HR to BCMV; white arrows indicating the vein necrosis of primary leaves on inoculation with BCMNV at temperature < 30°C. Susceptible plants (IC-328558) do not possess *I* gene for resistance.

BCMV and BCMNV isolates induced typical mosaic, necrosis, mottling, leaf crinkling and deformations in susceptible cultivars and accurately distinguished the resistant genotypes carrying the dominant “*II*” gene from the susceptible “*ii*” genotypes. Of all the 123 plant materials screened for BCMNV resistance, 13 genotypes presented localized and systemic vein necrosis that suggested the presence of “*I*” gene conditioning resistance through “Temperature Insensitive Necrosis (TIN)” in bean plants (Figure 1b, Supplementary File S1). The tested BCMNV isolate induced initial necrotic lesions on resistant plant materials that appeared at 4 dpi and expanded quickly, reaching the entire leaf veins and a nervure necrotic vein reaction was noticed at 10–12 dpi (Figure 1b, Supplementary File S1). Similarly, from the tested 123 plant materials, 23 genotypes conferred resistance to BCMV. However, infection with BCMV didn’t induce TIN in those genotypes conditioning HR to BCMNV and conferring extreme immunity (Figure 1a). Also, few genotypes induced mild necrosis of primary leaves infected with BCMV, demonstrating the incomplete dominant nature of the *I* gene as described by Collmer et al. (2000), that *I/i* genotypes respond to BCMV infection also through an HR.

**FIGURE 2:**
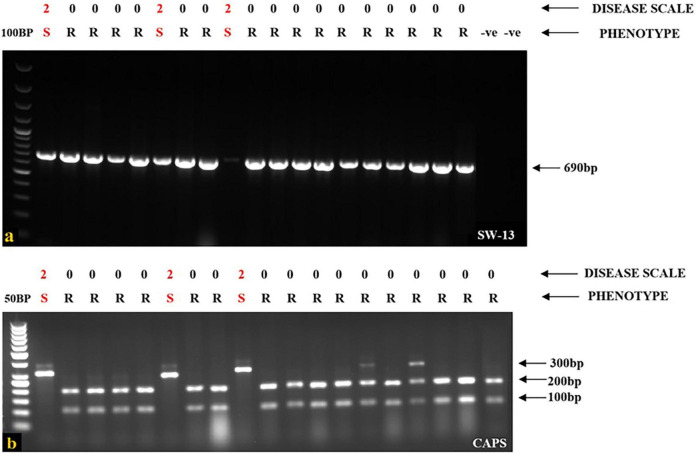
Identification of *I* gene for resistance in the screened genotypes. **(a)** SW13 marker identified hypersensitive resistant genotypes bearing *I* gene in which 3 cultivars are still susceptible with a disease scale of “2.” **(b)** CAPS marker identified and cleaved only resistant genotypes, whereas susceptible genotypes remain un-cleaved by *Taq*1. This marker yields two alleles. The resistant allele has two bands (200 and 100 bp), whereas the susceptible allele has a single band (311 bp). For both SW13 and CAPS markers, Lane 1–20, 1: Ladder (100 bp for SW13 and 50 bp for CAPS), 2 (EC-400444), 3 (EC-325065), 4(EC-400439), 5 (EC-13100), 6 (EC-127645), 7 (EC-271540), 8 (EC-405209), 9 (EC-116117), 10 (WB-6), 11 (WB-352), 12 (SR-3), 13 (WB-353 F), 14 (WB-N4), 15 (WB-N1), 16 (Local-2), 17 (Shalimar French bean-1), 18 (NSP-F1), 19 (NSP-F2), 20 (EC-325078), Lane 21, 22: Negative control for SW13 marker (DNA template from resistant plants).

**FIGURE 3:**
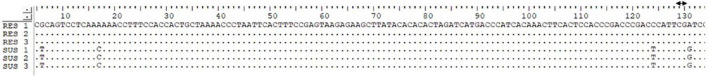
Illustrating sequence alignment of the *I* gene as identified by CAPS marker reveals four single-nucleotide polymorphisms (SNPs) that differentiate resistant and susceptible genotypes. A *Taq*I restriction site (TCGA) is present at nucleotide positions 126–130 in resistant genotypes, whereas a G-to-A substitution at position 128 results in the absence of this site in susceptible genotypes.

When a SCAR marker SW13 was utilized to screen all the genotypes for identifying the *I* gene, the marker consistently identified the plant materials presenting localized vein necrosis on the inoculated leaf. SW13 marker linked to the *I* gene amplified a 690 bp product from 16 genotypes (Table 2) that specified the presence of the dominant *I* gene for resistance (Figure 2a). However, three genotypes (EC-400444, EC-271540, and WB-6, Table 2), identified by the marker were susceptible to virus inoculation. They were later found to have mutations in the *I* gene at four nucleotide positions (Figure 3). To overcome these mutated forms, another co-dominant marker (BCMV-CAPS, Table 1) linked to the *I* gene was utilized. This marker is based on the presence of single nucleotide difference (A/G) between *I* gene-bearing resistant (*II*) and susceptible (*ii*) plants. Resistant plants have “A” allele while susceptible plants have “G” allele. PCR products (311 bp) of resistant genotypes on digestion with *Taq*I (Restriction site: TCG**A**) generated products of 201 and 110 bp, whereas susceptible plants remain un-cleaved (311 bp) by *Taq*I (Figure 2b) due to point mutations and absence of the restriction site (TCG**A**-TCG**G**). CAPS analysis based on these closely linked SNPs identified only true resistant plants bearing the *I* gene for resistance. PCR products from resistant and susceptible genotypes, containing the *I* gene corresponding to CAPS marker, were purified and sanger sequenced. Sequence alignment revealed point mutations between resistant and susceptible plants. One of these mutations created a *Taq*1 restriction site, as illustrated in Figure 3.

**TABLE 2:** Genotypes screened for resistance, phenotyping and resistance gene results.

S. N	Genotype	Disease rating	SW13 (*I* gene)	CAPS-BCMV (*I* gene)	ROC11 (*bc-3* gene)	ENM-F/R (*bc-3* gene)	Resistant phenotypes	
							**BCMV**	**BCMNV**
1.	EC-400444	1	** +**	−	+		−	−
2.	EC-400454	2	−	−	+		−	−
3.	EC-398512	2	−	−	+		−	−
4.	EC-405217	1	−	−	+		−	−
5.	EC-271555	3	−	−	+		−	−
6.	EC-18133	1	−	−	+		−	−
7.	EC-400453	2	−	−	+		−	−
8.	EC-271489	2	−	−	+		−	−
**9.**	**EC-325065**	**0**	** +**	**+**	**+**		**NVN**	**VN**
**10.**	**EC-405210**	**0**	−	−	+		**NVN**	**NVN**
11.	EC-398577	2	−	−	+		−	−
**12.**	**EC-24946**	**0**	−	−	−	−	**NVN**	**NVN**
13.	EC-400428	2	−	−	+		−	−
14.	EC-258279	3	−	−	+		−	−
15.	EC-397824	1	−	−	+		−	−
**16.**	**EC-400439**	**0**	** +**	**+**	**+**		**NVN**	**VN**
17.	EC-285579	2	−	−	+		−	−
**18.**	**EC-13100**	**0**	** +**	**+**	−	** +**	**NVN**	**NVN**
19.	EC-199205	2	−	−	+		−	−
**20.**	**EC-127645**	**0**	** +**	**+**	−	** +**	**NVN**	**NVN**
21.	EC-271540	1	+	−	+		−	−
22.	EC-286071	1	−	−	+		−	−
23.	EC-400408	2	−	−	+		−	−
**24.**	**EC-405209**	**0**	** +**	**+**	−	−	**MVN**	**VN**
**25.**	**EC-271544**	**0**	−	−	−	** +**	**NVN**	**NVN**
26.	EC-324976	2	−	−	+		−	−
**27.**	**EC-385259**	**0**	−	−	−	** +**	**NVN**	**NVN**
28.	EC-24956	3	−	−	+		−	−
29.	IC-328558	3	−	−	+		−	−
30.	IC-049696	2	−	−	+		−	−
31.	IC -049559	2	−	−	+		−	−
32.	IC-258259	2	−	−	+		−	−
33.	IC-262831	2	−	−	+		−	−
34.	IC-328896	2	−	−	+		−	−
35.	IC-041665	2	−	−	+		−	−
36.	EC-398567	2	−	−	+		−	−
**37.**	**EC-116117**	**0**	** +**	**+**	−	** +**	**NVN**	**NVN**
38.	EC-405193	2	−	−	+		−	−
39.	EC-28555	3	−	−	+		−	−
40.	IC-437141	3	−	−	+		−	−
41.	IC-381013	2	−	−	+		−	−
42.	IC-039073	2	−	−	+		−	−
43.	IC-37150	2	−	−	+		−	−
44.	IC-329154	1	−	−	+		−	−
45.	IC-041650	2	−	−	+		−	−
46.	IC-037137	2	−	−	+		−	−
47.	EC-400450	1	−	−	+		−	−
48.	EC-405208	2	−	−	+		−	−
49.	EC-271530	2	−	−	+		−	−
50.	EC-316026	2	−	−	+		−	−
51.	EC-398500	1	−	−	+		−	−
52.	EC-942386	2	−	−	+		−	−
**53.**	**EC-325078**	**0**	** +**	**+**	**+**		**MVN**	**VN**
**54.**	**WB-1137**	**0**	−	−	−	** +**	**NVN**	**NVN**
**55.**	**WB-1670**	**0**	−	−	−	** +**	**NVN**	**NVN**
56.	WB-846	2	−	−	** +**		−	−
57.	KRC-22	1	−	−			−	−
58.	SKAU-R-91	2	−	−			−	−
59.	WB-662	2	−	−			−	−
**60.**	**WB-1316**	**0**	−	−	−	−	**NVN**	**NVN**
61.	836	2	−	−			−	−
62.	SR 2	1	−	−			−	−
63.	WB-222	1	−	−			−	−
64.	WB-1304	2	−	−			−	−
**65.**	**WB-352**	**0**	** +**	**+**	**+**	**-**	**MVN**	**VN**
66.	WB-920	2	−	−			−	−
67.	1677	2	−	−			−	−
68.	22721	1	−	−			−	−
69.	N2	1	−	−			−	−
70.	WB-369	3	−	−			−	−
71.	WB-195	2	−	−			−	−
72.	WB-59	2	−	−			−	−
73.	WB-1678	1	−	−			−	−
**74.**	**SR-3**	**0**	** +**	**+**	**-**	**-**	**NVN**	**VN**
75.	WB-1129	3	−	−			−	−
76.	WB-22	1	−	−			−	−
77.	WB-216	2	−	−			−	−
78.	WB-112	1	−	−			−	−
**79.**	**WB-352 (field)**	**0**	** +**	**+**	**+**		**NVN**	**VN**
**80.**	**WB-N4**	**0**	** +**	**+**	**-**	**-**	**NVN**	**VN**
**81.**	**WB-N1**	**0**	** +**	**+**	**-**	**-**	**MVN**	**VN**
82.	**WB-6**	**1**	** +**	−	+		−	−
83.	ALR-6	1	−	−	+		−	−
84.	ALR-31	2	−	−	+		−	−
85.	ALR-59	2	−	−	+		−	−
86.	ALR-49	1	−	−	+		−	−
87.	ALR-54	3	−	−	+		−	−
88.	ALR-66	3	−	−	+		−	−
89.	ALR-94	3	−	−	+		−	−
90.	ALR-1	2	−	−	+		−	−
91.	ALR-50	2	−	−	+		−	−
92.	ALR-56	1	−	−	+		−	−
93.	ALR-62	1	−	−	+		−	−
94.	ALR-34	3	−	−	+		−	−
95.	ALR-79	2	−	−	+		−	−
96.	ALR-78	3	−	−	+		−	−
97.	ALR-70	1	−	−	+		−	−
98.	ALR-8	2	−	−	+		−	−
99.	ALR-13	3	−	−	+		−	−
100.	ALR-90	1	−	−	+		−	−
101.	ALR-82	2	−	−	+		−	−
102.	ALR-5	1	−	−	+		−	−
103.	ALR-76	3	−	−	+		−	−
104.	Local A	3	−	−	+		−	−
105.	Local B	2	−	−	+		−	−
106.	Local C	1	−	−	+		−	−
107.	Local-1	1	−	−	+		−	−
**108.**	**Local-2**	**0**	** +**	**+**	+		**NVN**	**VN**
109.	Local-3	2	−	−	+		−	−
110.	1129	2	−	−	+		−	−
111.	N-15	3	−	−	+		−	−
112.	Arka Komal	2	−	−	+		−	−
**113.**	**Shalimar French bean-1**	**0**	** +**	**+**	+		**NVN**	**VN**
114.	SR-1	3	−	−	+		−	−
115.	SR-4	1	−	−	+		−	−
116.	SR-101	2	−	−	+		−	−
117.	SKAU NSP-2	3	−	−	+		−	−
118.	SKAU NSP-3	2	−	−	+		−	−
119.	SKAU NSP-4	2	−	−	+		−	−
120.	SR-2	2	−	−	+		−	−
121.	SKAU-5	1	−	−	+		−	−
**122.**	**NSP F-1**	**0**	** +**	**+**	+		**NVN**	**VN**
**123.**	**NSP F-2**	**0**	** +**	**+**	+		**NVN**	**VN**

**VN:** Resistant Genotypes that show Vein Necrosis (VN) on the inoculated leaves. **NVN:** Resistant Genotypes that display No Vein Necrosis (NVN) on the inoculated leaf. **MVN:** Resistant Genotypes that display Mild Vein Necrosis (MVN) on the inoculated leaf. **SW13**: “+” denotes presence of *I* gene as identified by the marker. “−” denotes absence of *I* gene as identified by the marker. **BCMV-CAPS**: “ + “ denotes presence of *I* gene after digestion of PCR amplicon (311 bp) with *Taq*I that generates products of 201 and 110 bp. “−” denotes absence of *I* gene after digestion of PCR amplicon (311 bp) with *Taq*I, which does not generate products of 201 and 110 bp and remains un-cleaved (311 bp). **ROC11:** “+” denotes **absence** of *bc-3* gene. “−” denotes the **presence** of the *bc-3* gene. **ENM-F/R: “+**” denotes presence of two signals (381 and 160 bp) after digestion with *Rsa*1 (resistant). “−” denotes presence of undigested 541-bp fragment after digestion with *Rsa*1 (susceptible). For the ENM-F/R test, only genotypes identified as carrying the *bc-3* gene (using the ROC11/420 marker) were used.

### 3.3 Phenotypic and genotypic evaluation of plant materials bearing the *bc-3* gene

**FIGURE 4:**
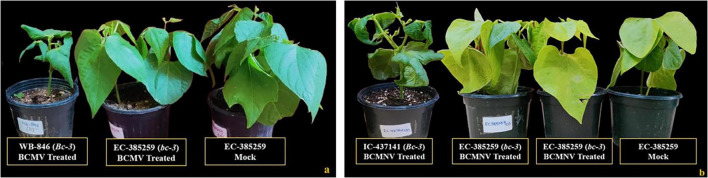
Resistant genotypes identified for the presence of the recessive “*bc-3*” gene. **(a)** All treatments inoculated with BCMV and a mock (EC-385259, conditioning resistance to BCMV). **(b)** All treatments inoculated with BCMNV and a mock (EC-385259, conditioning resistance to BCMNV). Susceptible plants (WB-846 and IC-437141) do not possess *bc-3* gene for resistance.

This resistant group included 4 genotypes that were resistant and symptomless after mechanical inoculation (BCMV and BCMNV) and were tested for the presence of the “*bc-3*” gene in PCR assays. Genotypes carrying this recessive resistance allele conferred complete immunity to both BCMV and BCMNV isolates (Figures 4a,b), without triggering any necrotic reaction on the inoculated leaves. Plant materials with the *bc-3* gene never reacted with mosaic or necrosis and the only visible reaction was necrotic, in which pin-point necrotic spots developed on upper un-inoculated leaves in between 10 and 15 dpi infected with BCMNV (Figure 4) which were less prominent and did not extend into localized vein necrosis. No systemic necrosis can be exemplified as the plant materials are visibly healthy and recorded no symptoms throughout the 30-day phenotyping period.

The presence of the *bc-3* gene in this group was confirmed by both ROC11 and ENM-FWe/Rve markers. The absence of SCAR marker ROC11/420 indicates the presence of *bc-3* gene and its negative selection was initially used to eliminate the common bean genotypes lacking *bc-3* disease resistance locus (Table 2). Subsequently, those genotypes bearing the *bc-3* gene as identified by ROC11 marker were amplified using ENM-FWe/Rve marker. The genotypes which were not identified for the presence of *bc-3* gene using ROC11 marker were not utilized to test with ENM-FWe/Rve marker. Digestion of PCR amplified products (541-bp fragment) with *Rsa*I cleaved *bc-3* carrying genotypes into 381- and 160-bp fragments, whereas the PCR products derived from the susceptible genotypes remain un-cleaved, due to the absence of mutations within the *eIF4E* gene. To confirm the presence of mutations in the *eIF4E* gene of resistant genotypes, the 541-bp PCR products were purified and sanger sequenced. Pairwise nucleotide sequence comparison was performed by using the Basic Local Alignment Search Tool (BLAST) and the partial coding sequence (541 bp) of genotypes carrying the *bc-3* gene had a maximum identity of 100% to the published *eIF4E* gene sequence of the cultivar “IVT7214” (KT175572), reported to carry *bc-3* gene.

### 3.4 Phenotypic and genotypic evaluation of plant materials bearing *I* and *bc-3* gene

**FIGURE 5:**
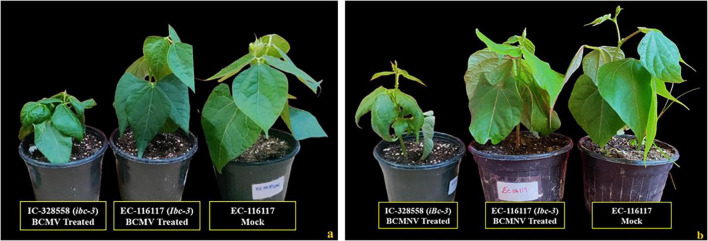
Resistant genotypes identified for the presence of both “*I*” and “*bc-3*” gene. **(a)** All treatments inoculated with BCMV and a mock (EC-116117, conditioning resistance to BCMV). **(b)** All treatments inoculated with BCMNV and a mock (EC-116117, conditioning resistance to BCMNV). Susceptible plants (IC-328558) do not possess both the *I* and *bc-3* genes for resistance.

Three genotypes (EC-13100, EC-127645, EC-116117) bearing both *I* and *bc-3* (*Ibc-*3) genes for resistance were identified from the screened germplasm. These genotypes presented no symptoms or any HR (Figures 5a,b) on both inoculated and upper un-inoculated leaves to both BCMV and BCMNV isolates. Hence, these genotypes were protected from BCMNV-induced necrosis and conditioned extreme resistance to the bean plants without subjecting them to HR. In this gene combinations (*Ibc-3*), the dominant *I* gene was protected from being necrotic by the presence of the *bc-3* allele.

**FIGURE 6:**
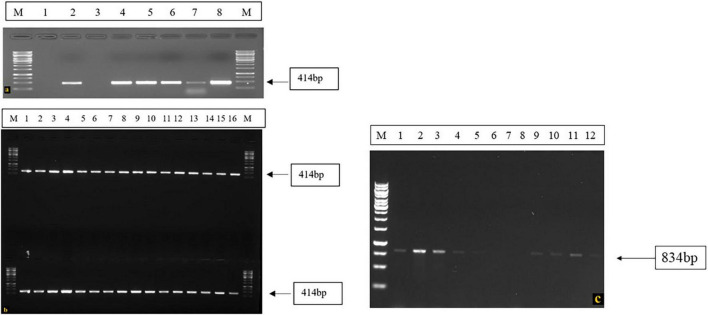
RT-PCR confirmation of presence of virus in the symptomatic and susceptible genotypes using BCMV-BCMNV CP primers, **(a)** M: 1 kb DNA ladder, 1 and 3: Negative Controls (RNA isolated from Resistant Plants), 2: BCMV Positive control, 4–8: RNA isolated from BCMV susceptible genotypes. **(b)** M: 1 kb DNA ladder, 1–16: BCMV Susceptible genotypes. **(c)** RT-PCR amplification of BCMNV in susceptible genotypes, Lane M: 1 kb DNA ladder, Lane 1: BCMNV positive control, Lane 2–5: Susceptible genotypes, Lane 6–8: Resistant genotypes (Negative control), Lane 9–12: Susceptible genotypes.

However, 3 resistant plant materials in which both *I* and *bc-3* genes were not identified, the presence of other resistance genes or gene combination can be assumed, which protects the common beans against BCMV-BCMNV. Due to its minor influence on conferring resistance to the virus and also due to the limited availability of markers linked to these genes, its presence in the screened germplasm was not established. These genotypes conferred high resistance to the test isolates and the viral symptoms didn’t appear on both inoculated and upper uninoculated leaves till the entire phenotyping period of 30 dpi. In susceptible plants, the RT-PCR assay using BCMV and BCMNV primers yielded positive results, and did not detect the presence of the virus in resistant plants (Figure 6). Susceptible plants associated with the symptoms of mosaic, necrosis, leaf crinkling and puckering were amplified around 414 bp with BCMV CP primers and no BCMV presence was detected in resistant genotypes (Figures 6a,b). Whereas, BCMNV primers amplified the target virus with expected product size of 834 bp from susceptible genotypes (Figure 6c) and no BCMNV was detected from the RNA isolated from resistant genotypes.

### 3.5 qPCR-based assessment of virus accumulation

**FIGURE 7:**
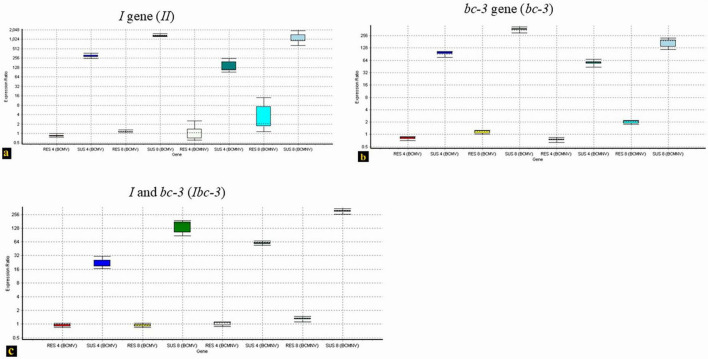
RT-qPCR based comparative quantification on the accumulation of BCMV-BCMNV in three resistant genotypes, **(a)** WB-352 (*I*). **(b)** EC-127645 (*bc-3*). **(c)** EC-116117 (*Ibc-3)* at two time points (Day 4 and Day 8). In three experiments **(a–c)**, plants were inoculated with BCMV and BCMNV and its expression in the top leaves was analyzed in qPCR assay. The expression ratios of BCMV-BCMNV in both resistant and susceptible groups are represented by Whisker box plots and are calculated using the take-off and replication values of control plants (untreated) against treated group (resistant and susceptible plants) in REST software (QIAGEN).

Resistant phenotypes of bean cultivars differed based on the presence or absence of the *I* gene. The two test isolates induced either local or systemic necrosis in resistant cultivars (WB-352) carrying the *I* gene, but not in those genotypes carrying *Ibc-3* (EC-116117) and *bc-3* (EC-127645) resistant genes. qPCR analysis of the RNA isolated from the upper leaves of three different genotypes (*I, bc-3, and Ibc-3*) at the 4th and 8th day post-inoculation revealed varying levels of BCMV-BCMNV expression across the genotypes. Cultivars carrying the *I* gene alone exhibited low-level viral expression on Day 4, where initial necrotic lesions appeared on the inoculated leaves and an increased expression at day 8 (Figure 7a) that corresponds to extensive vein necrosis that usually appears at 10 dpi. In contrast, no viral spread was detected in *Ibc-3* (Figure 7c) and *bc-3* (Figure 7b) genotypes up to 8 dpi, and these plants remained visibly healthy throughout the 30-day observation period. This suggests that genotypes carrying the *I* gene alone would enable the virus to replicate and allow for systemic movement inside the host plants without the symptoms being expressed that were affected in the other genotypes (*Ibc-3, bc-3*), where its replication and movement were inhibited. The melting and amplification curves of the tested genotypes were provided in Supplementary File S2.

## 4 Discussion

BCMV and BCMNV are two closely related viruses that pose a significant threat to global common bean production. These viruses can cause complete crop failure (up to 100% yield loss) and significantly reduce bean quality (Tang and Feng, 2022; Worrall et al., 2015). In response, identifying and developing common bean cultivars with broad genetic resistance to these viruses is crucial.

In this study, 123 common bean genotypes were inoculated to assess resistance and susceptibility to BCMV-BCMNV and to identify the plant materials bearing multiple resistant genes, each working with a different mode of action. Two forms of resistance were observed in the identified plant materials: some exhibited a hypersensitive reaction (HR) to the virus, while others remained resistant without exhibiting HR. Resistant plants exhibiting HR presented a vein necrosis of primary inoculated leaves between 7 and 10 dpi. Phenotypically, this necrotic vein reaction of inoculated primary leaves was the key feature in demonstrating the presence of the *I* gene (Feng et al., 2017; Collmer et al., 2000). This necrotic sign has been identified in most of the resistant cultivars (13 genotypes) in the screened germplasm and conferred resistance toward BCMV-BCMNV under controlled conditions (< 30°C). The evidence for the presence of the *I* gene was suggested by PCR using SW-13 and BCMV-CAPS markers. These results are in accordance with some previous studies that the *I* gene confers a high degree of resistance and complete immunity to BCMV strains below 30°C (Ali, 1950; Vallejos et al., 2006; Feng et al., 2017) and induces a necrotic reaction on primary inoculated leaves to BCMNV, regardless of the temperature. However, the wide use of common bean genotypes bearing the *I* gene has led to BCMNV-induced systemic necrosis and in response, its susceptibility can be prevented if the *I* gene is “protected” in the presence of one of the recessive genes (*bc-1, bc-2, or bc-3*) (Kelly et al., 1995; Singh and Schwartz, 2010; Pasev et al., 2014; Feng et al., 2018). We then focused on the presence of any recessive resistant gene in the remaining plant materials that were immune (without HR) to both BCMV and BCMNV. Interestingly, four resistant genotypes possessed the *bc-3* gene, and three genotypes were in combination of both *I* and *bc-3* genes (*Ibc-3*). However, some bean varieties with *bc-3* resistance to BCMNV were also reported as susceptible to BCMV (Miklas et al., 1998). The recessive *bc-3* gene encodes a mutated eukaryotic translation initiation factor (*eIF4E*) gene and was identified to be associated with resistance against BCMV-BCMNV in common beans. The VPg protein of the virus interacts with *eIF4E* proteins in plants and four-point mutations in the eIF4E gene impaired the interaction between *eIF4E* and VPg protein and also affected the replication of BCMV, thus conferring resistance in common beans (Naderpour et al., 2010). These amino acid polymorphisms in eIF4E were also reported previously to confer recessive resistance to *Potyviruses* in *Pisum sativum* against pea seed-borne mosaic virus, *Capsicum annuum* against chili veinal mottle virus and potato virus Y (PVY), *Hordeum vulgare* against barley yellow mosaic virus, *Citrullus lunatus* against zucchini yellow mosaic virus, *Lycopersicum esculentum* against potato virus Y (PVY) and tobacco etch virus (TEV) (Ashby et al., 2011; Hwang et al., 2009; Li et al., 2016; Ling et al., 2009; Piron et al., 2010; Ruffel et al., 2002; Ruffel et al., 2006).

A qPCR experiment was conducted to study the resistance conferred by the three resistant groups (*I, bc-3, Ibc-3*) that affect the systemic movement of the virus. This assay (Figure 7) also suggests that systemic spread of the virus was drastically affected in genotypes bearing *Ibc-3* genes with both BCMV and BCMNV being unable to replicate independently and spread to the uninoculated upper leaves. However, the genotypes bearing the *I* gene demonstrate a difference in virus spread in uninoculated upper leaves when compared to those genotypes bearing *bc-3* and *Ibc-3* genes. The systemic movement of virus in the bean genotypes carrying *I* alleles here demonstrated that BCMV replication occurs inside the host plants. It was also proven in the screening experiment that, bean genotypes carrying the *I* gene were immune to BCMV and a few genotypes also conditioned resistance to BCMV through a mild necrosis of primary leaves. Previous experiments with bean lines carrying *I* alleles also demonstrated that BCMV replication depends on the *I* allele’s dosage (Cadle-Davidson and Jahn, 2005) and the virus could still replicate in genotypes carrying the *I* gene even at a low temperature (26°C) (Collmer et al., 2000). This suggests that BCMV/BCMNV strains are capable of replicating and undergoing intercellular movement within *I*-gene-containing common bean cultivars without expressing any viral symptoms and necrosis likely occurs when the virus spreads from the initially infected cell to a neighboring cell (Collmer et al., 2000; Cadle-Davidson and Jahn, 2005; Feng et al., 2017). So, the best combination of genes in breeding for BCMV-BCMNV resistance was likely the *Ibc-3* combination as they confer a broad spectrum of genetic resistance (Miklas et al., 1998; Pasev et al., 2014; Singh and Schwartz, 2010; Worrall et al., 2015). This beneficial gene combination (*Ibc-3*) has been identified in some plant materials that provide promising resistance to BCMV-BCMNV also at temperatures above 30°C.

Hence, this study demonstrates the mechanism of resistance conferred by common bean cultivars bearing the dominant “*I*” and recessive “*bc-3*” genes and represents the influence of different genetic backgrounds on resistance to BCMV-BCMNV. Both SCAR (SW13 and ROC11) and CAPS (BCMV-CAPS and ENM-FWe/Rve) molecular markers used in this study were reliable for the identification of *I* and *bc-3* genes. Importantly, there are no reported instances of recombinant viral strains overcoming resistance conferred by these genes in India. In this respect, this investigation was important to separate common bean cultivars with major genes for resistance. We can further introduce these genes directly into our elite common bean varieties that are susceptible to BCMV and can later be utilized in bean breeding program for virus resistance.

## Data Availability

The original contributions presented in the study are included in the article/Supplementary material, further inquiries can be directed to the corresponding author.
