# Supplementary material for: Delineating the source of resistance to bean common mosaic virus (BCMV) and bean common mosaic necrosis virus (BCMNV) in common bean (Phaseolus vulgaris) cultivars of Jammu and Kashmir, a North-Western Himalayan region
Source: Front Microbiol. 2025 Jun 24;16:1614122. doi: 10.3389/fmicb.2025.1614122 (PMC12234503; doi:10.3389/fmicb.2025.1614122)
Supplement: Supplementary file 1 [file Supplementary_file_1.pptx]

## Slide 1
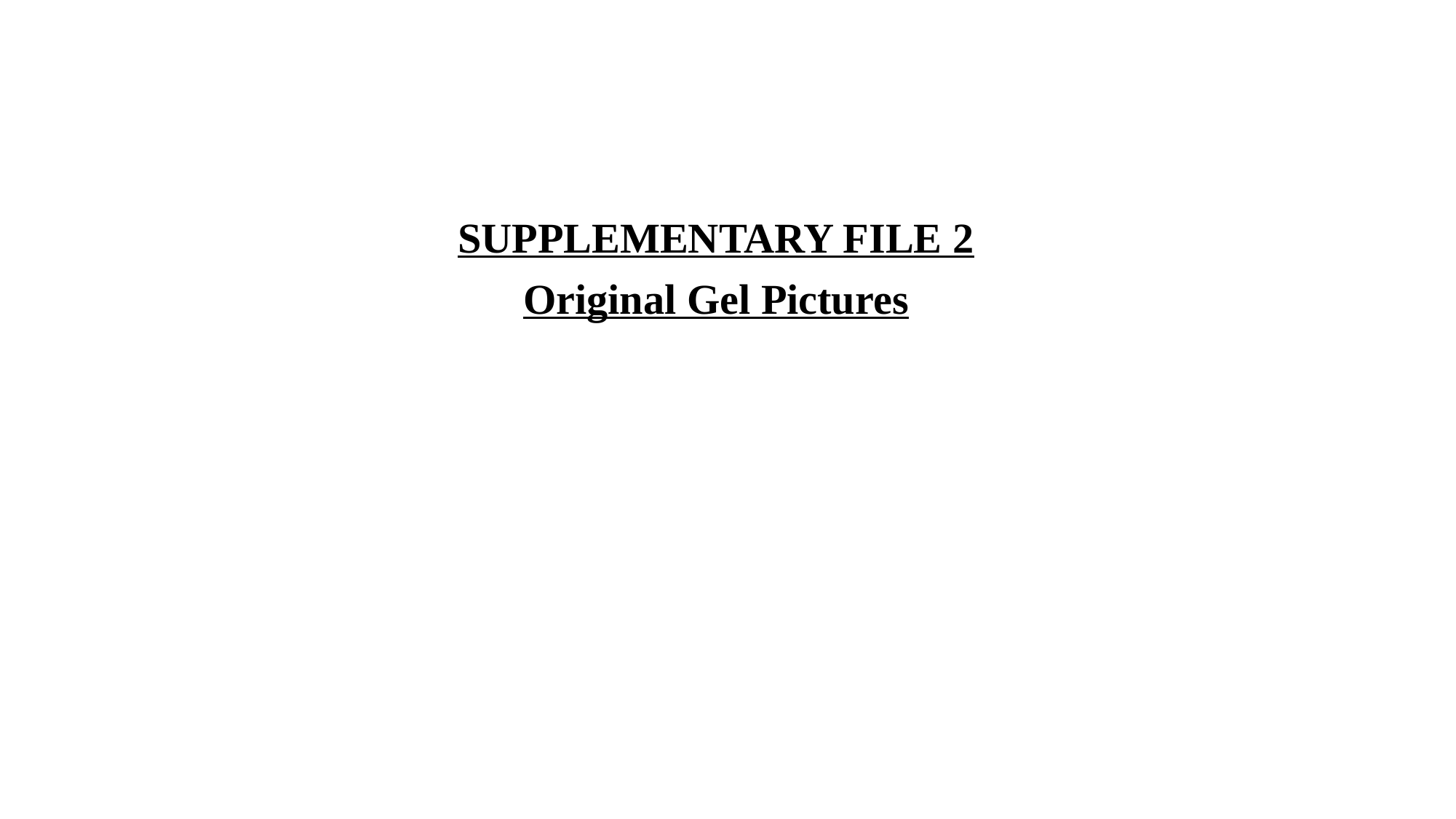

SUPPLEMENTARY FILE 2
Original Gel Pictures

## Slide 2
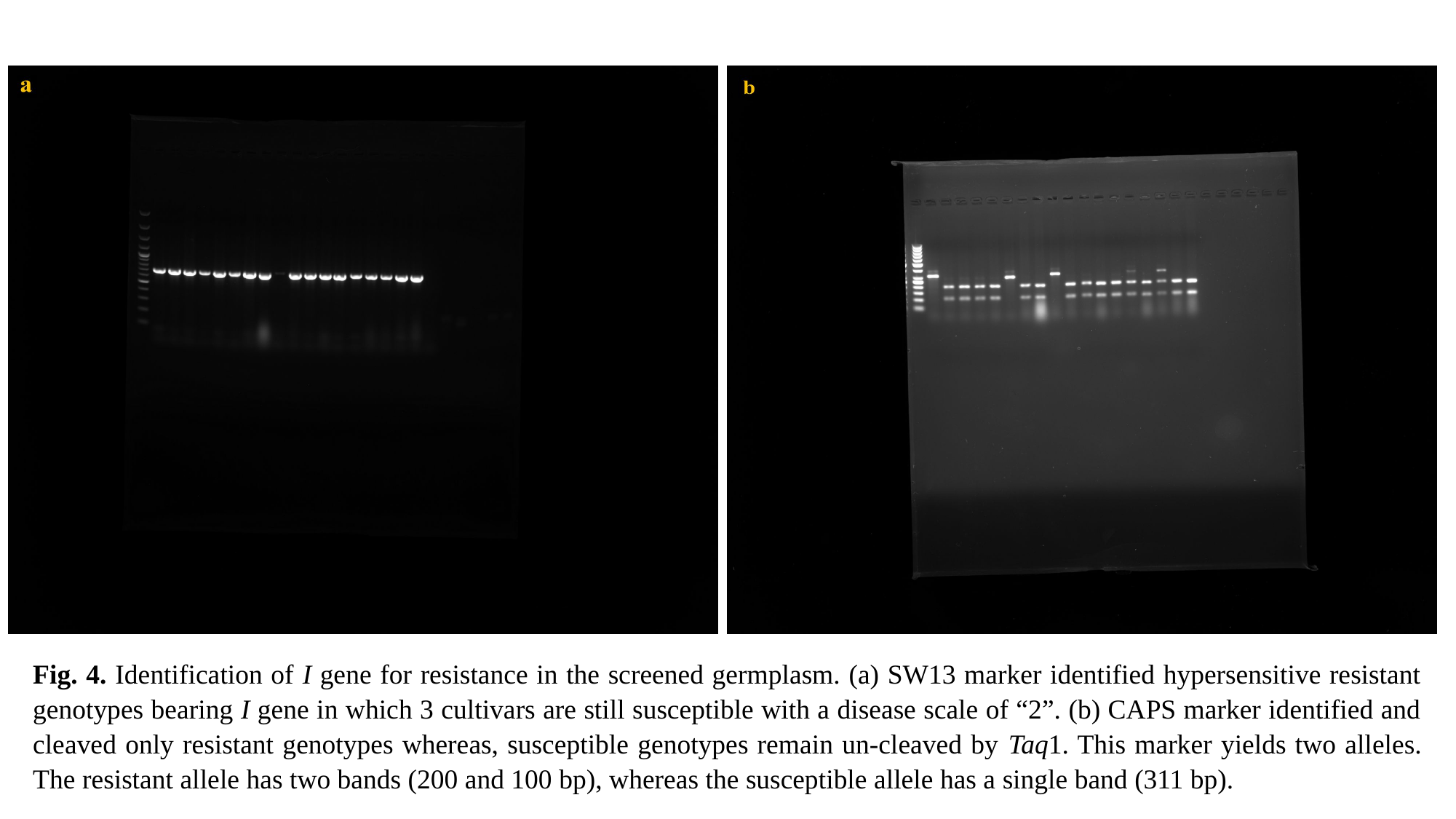

Fig. 4. Identification of I gene for resistance in the screened germplasm. (a) SW13 marker identified hypersensitive resistant genotypes bearing I gene in which 3 cultivars are still susceptible with a disease scale of “2”. (b) CAPS marker identified and cleaved only resistant genotypes whereas, susceptible genotypes remain un-cleaved by Taq1. This marker yields two alleles. The resistant allele has two bands (200 and 100 bp), whereas the susceptible allele has a single band (311 bp).

## Slide 3
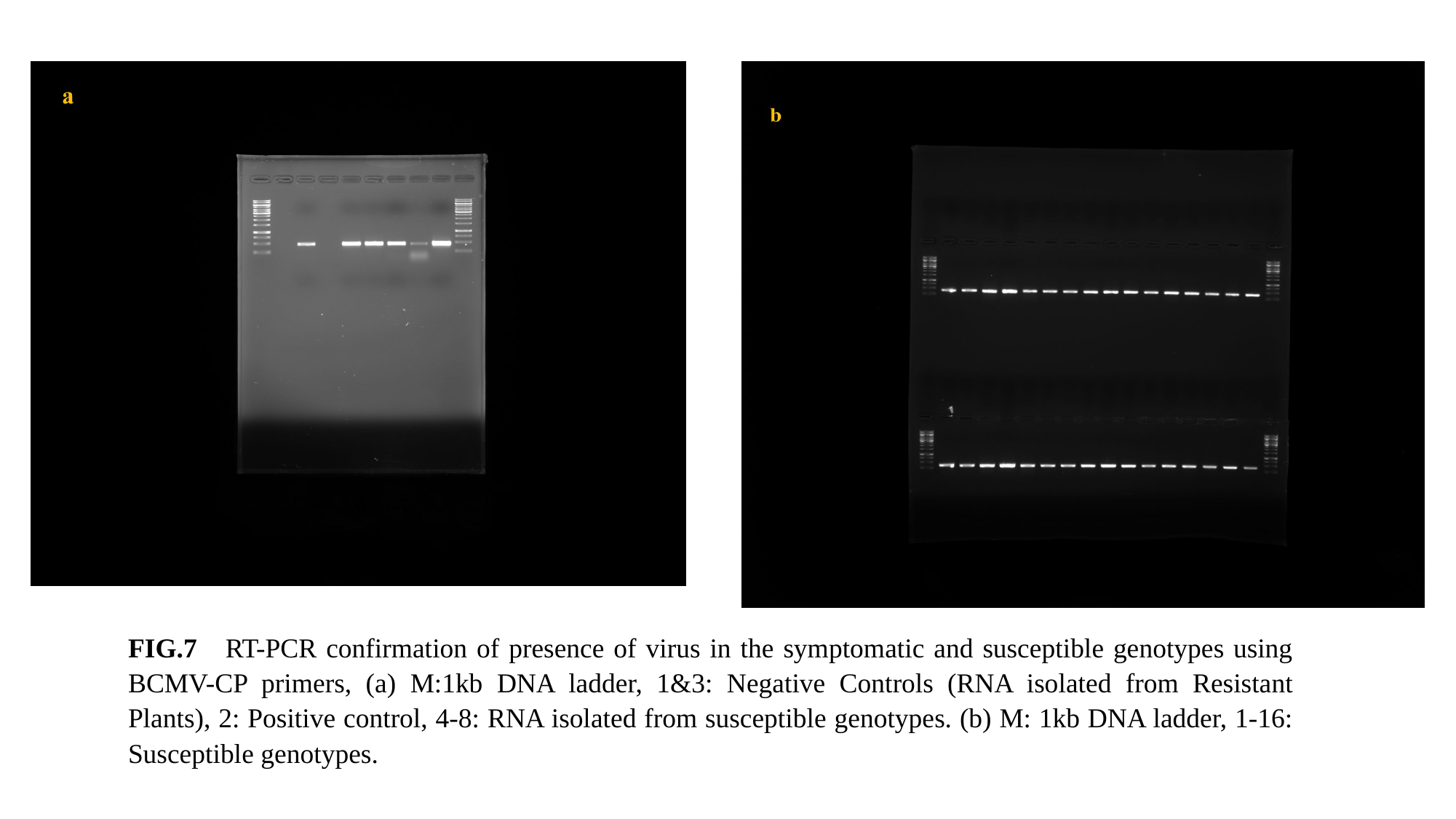

FIG.7 RT-PCR confirmation of presence of virus in the symptomatic and susceptible genotypes using BCMV-CP primers, (a) M:1kb DNA ladder, 1&3: Negative Controls (RNA isolated from Resistant Plants), 2: Positive control, 4-8: RNA isolated from susceptible genotypes. (b) M: 1kb DNA ladder, 1-16: Susceptible genotypes.

## Slide 4
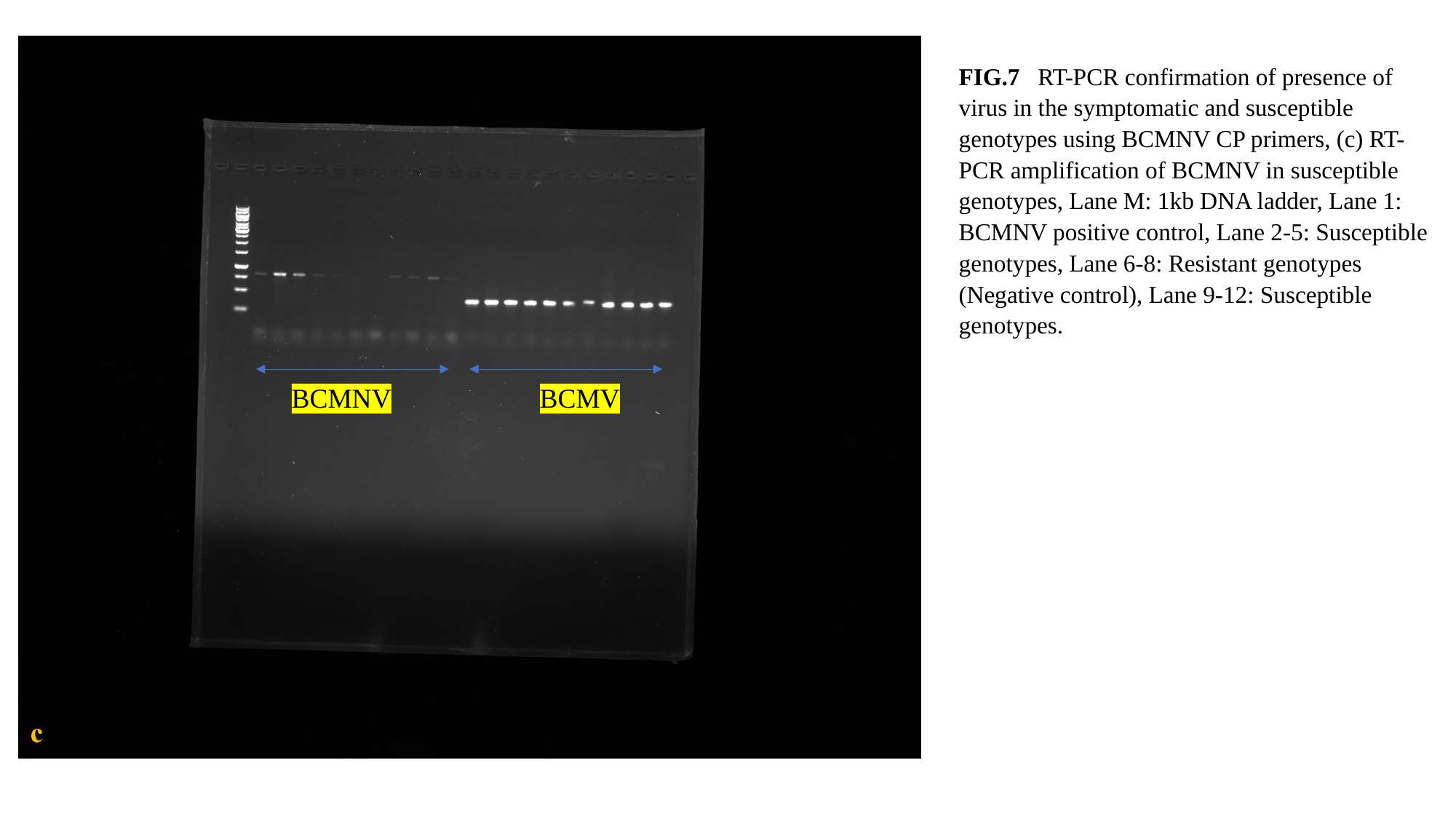

FIG.7 RT-PCR confirmation of presence of virus in the symptomatic and susceptible genotypes using BCMNV CP primers, (c) RT-PCR amplification of BCMNV in susceptible genotypes, Lane M: 1kb DNA ladder, Lane 1: BCMNV positive control, Lane 2-5: Susceptible genotypes, Lane 6-8: Resistant genotypes (Negative control), Lane 9-12: Susceptible genotypes.
BCMNV
BCMV

## Slide 5
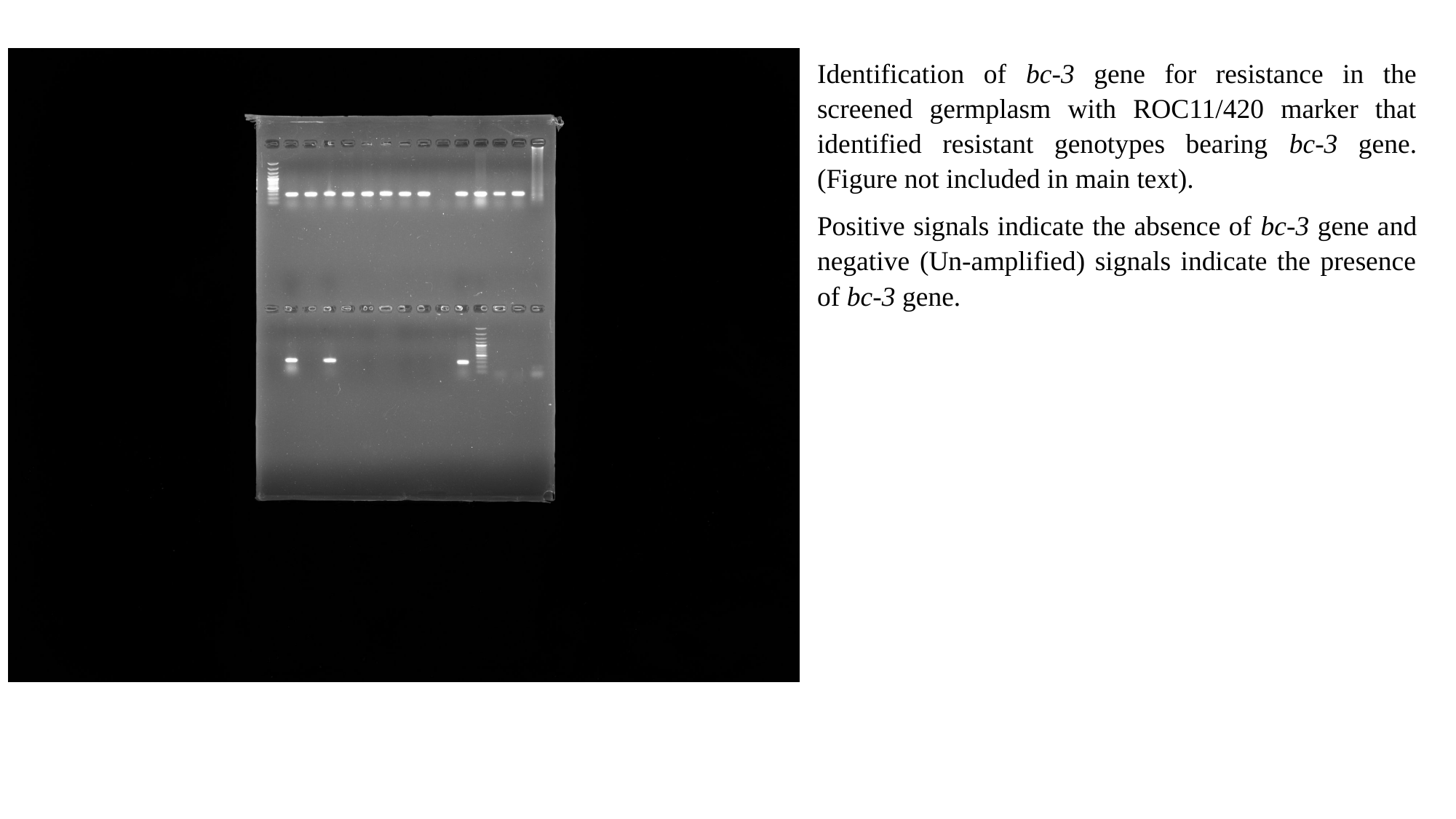

Identification of bc-3 gene for resistance in the screened germplasm with ROC11/420 marker that identified resistant genotypes bearing bc-3 gene. (Figure not included in main text).
Positive signals indicate the absence of bc-3 gene and negative (Un-amplified) signals indicate the presence of bc-3 gene.

## Slide 6
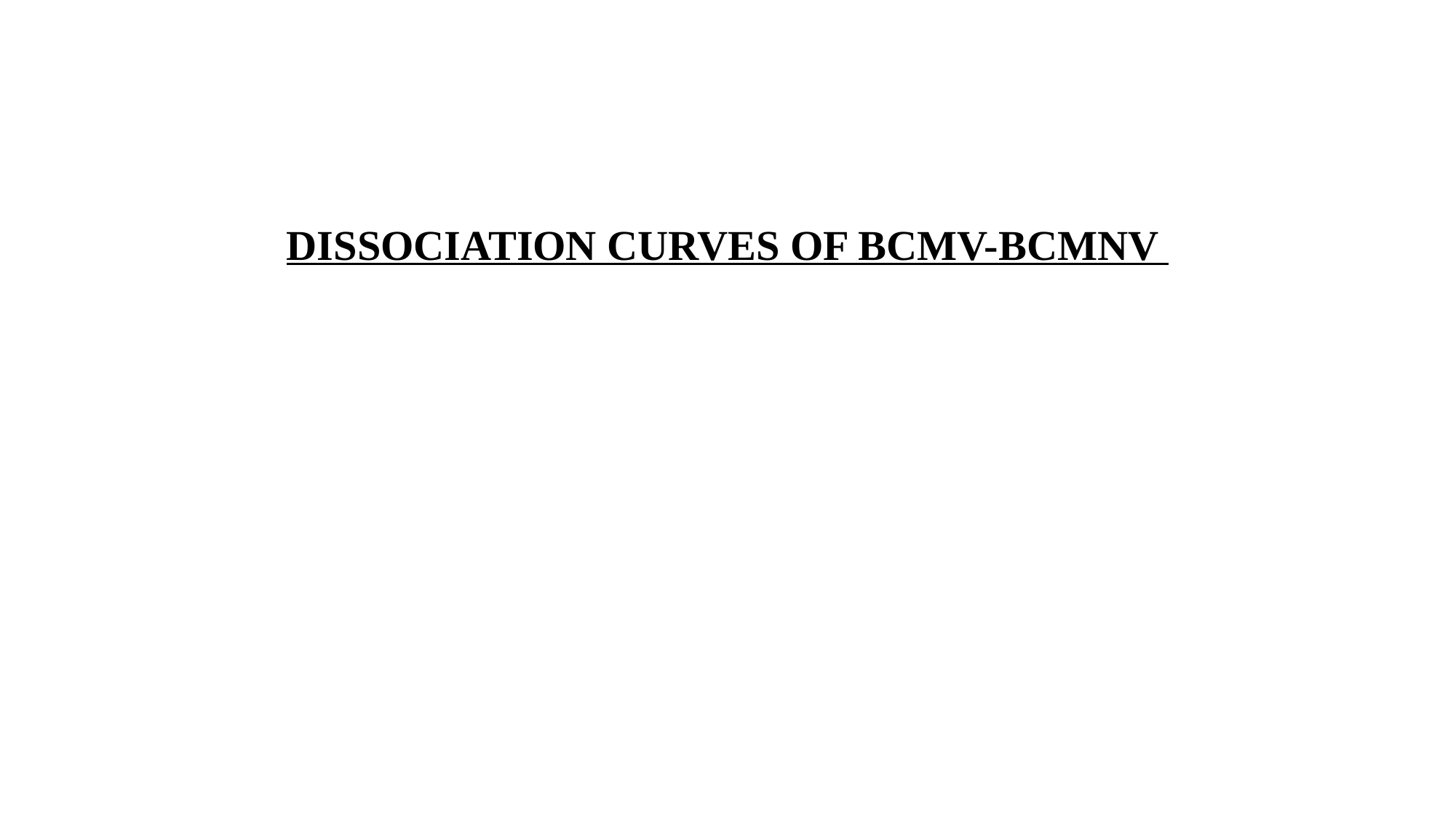

DISSOCIATION CURVES OF BCMV-BCMNV

## Slide 7
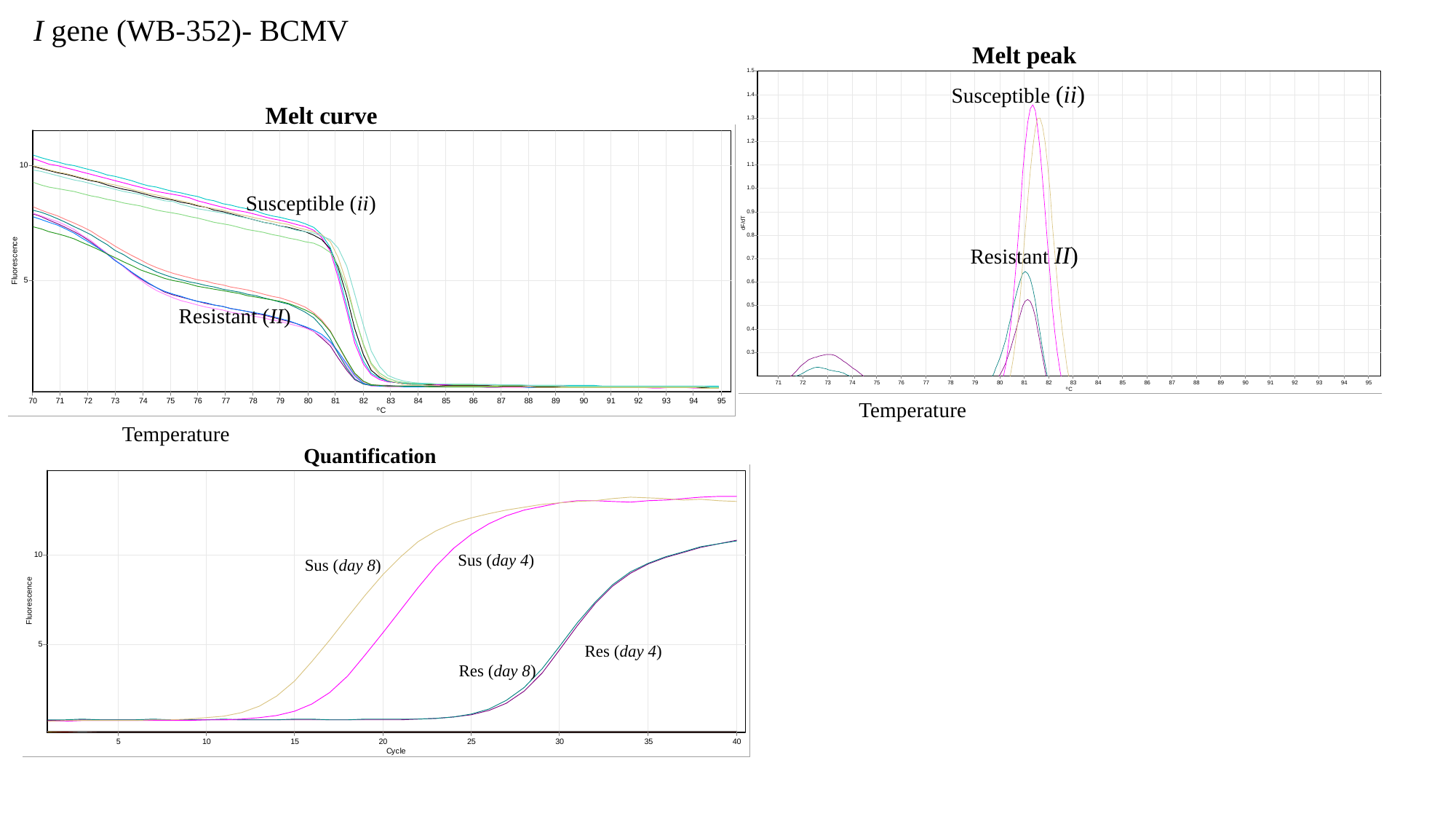

# I gene (WB-352)- BCMV
Melt peak
Susceptible (ii)
Melt curve
Susceptible (ii)
Resistant II)
Resistant (II)
Temperature
Temperature
Quantification
Sus (day 4)
Sus (day 8)
Res (day 4)
Res (day 8)

## Slide 8
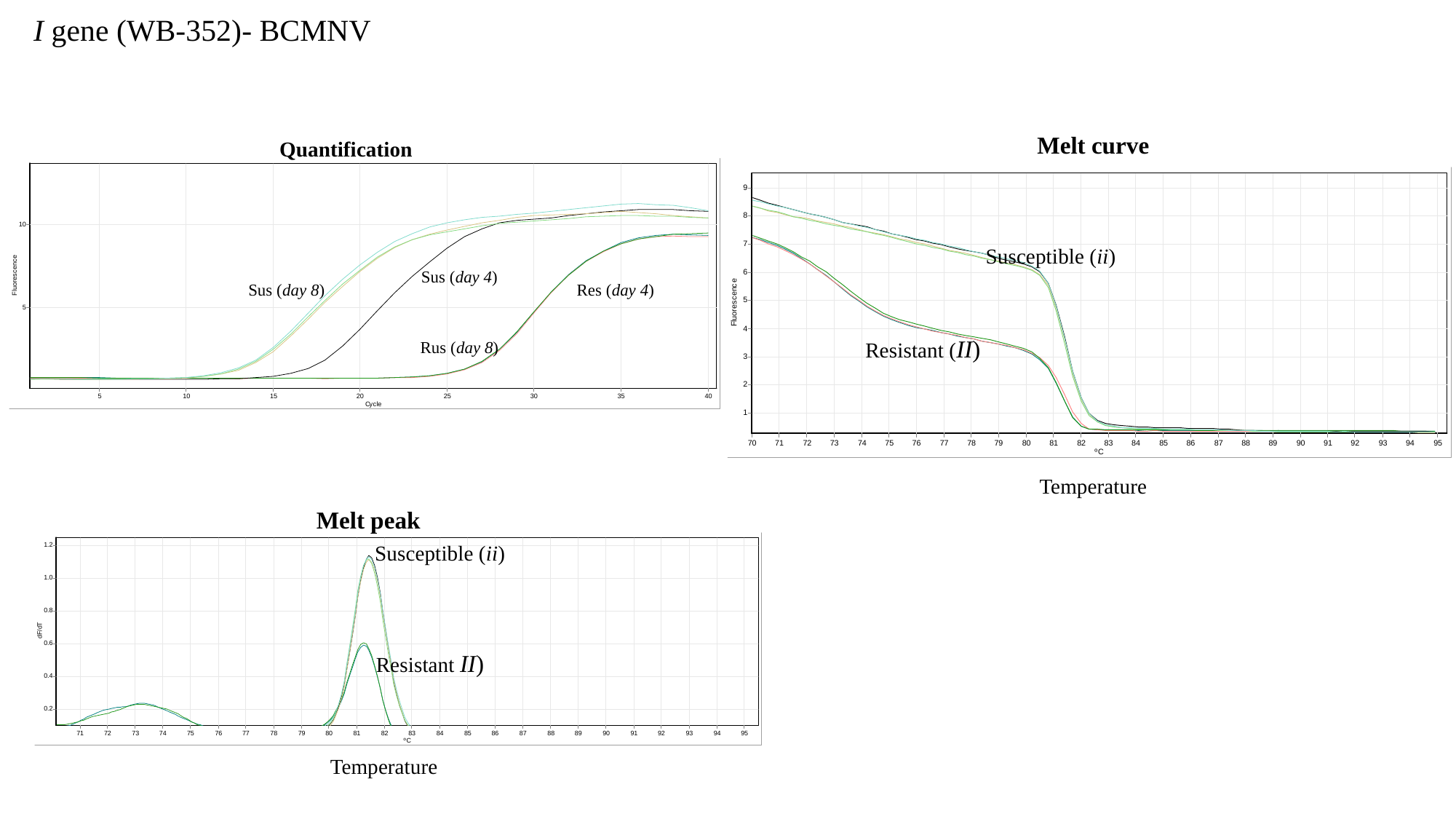

# I gene (WB-352)- BCMNV
Melt curve
Quantification
Susceptible (ii)
Sus (day 4)
Sus (day 8)
Res (day 4)
Resistant (II)
Rus (day 8)
Temperature
Melt peak
Susceptible (ii)
Resistant II)
Temperature

## Slide 9
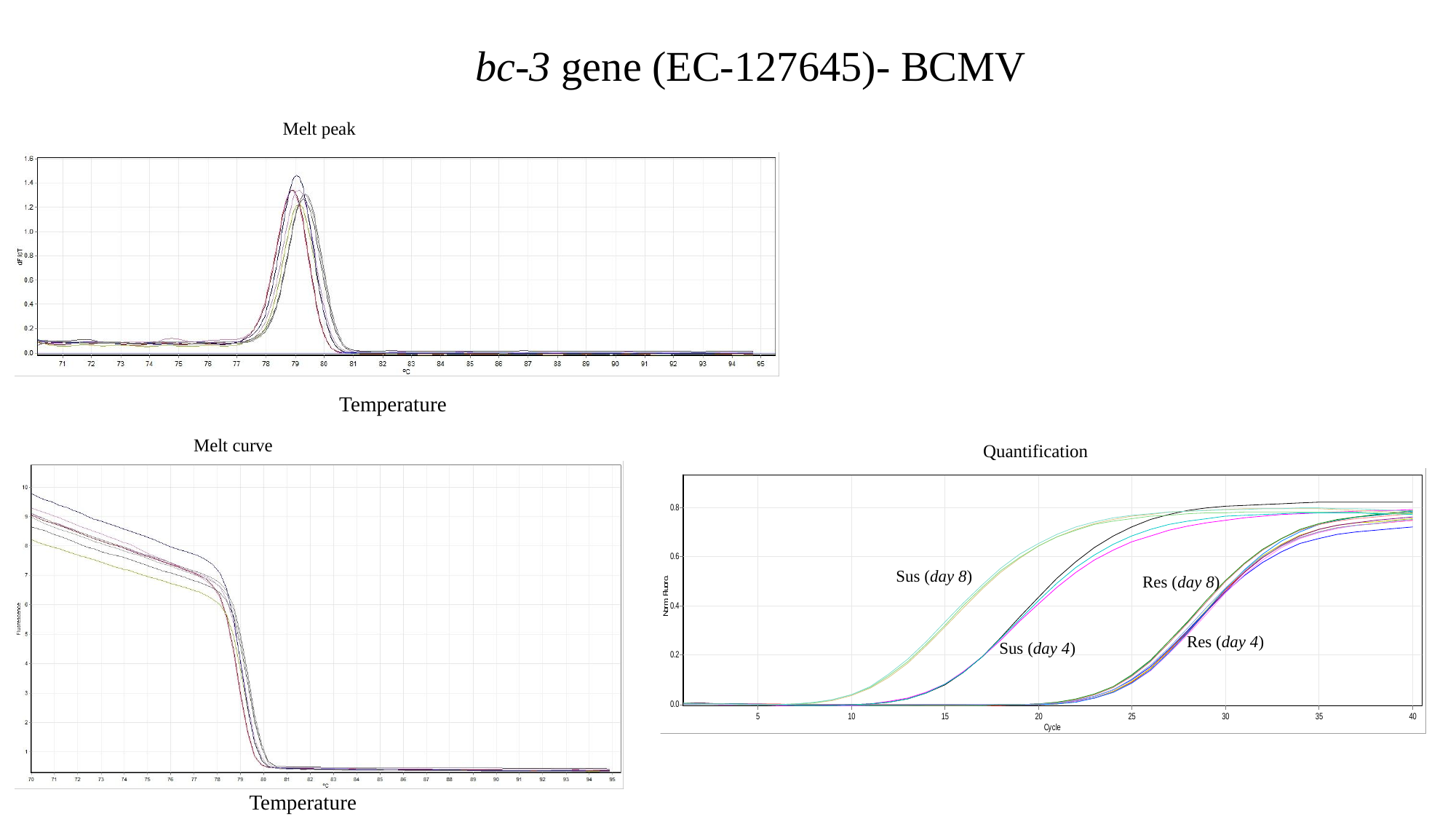

# bc-3 gene (EC-127645)- BCMV
Melt peak
Temperature
Quantification
Melt curve
Sus (day 8)
Res (day 8)
Res (day 4)
Sus (day 4)
Temperature

## Slide 10
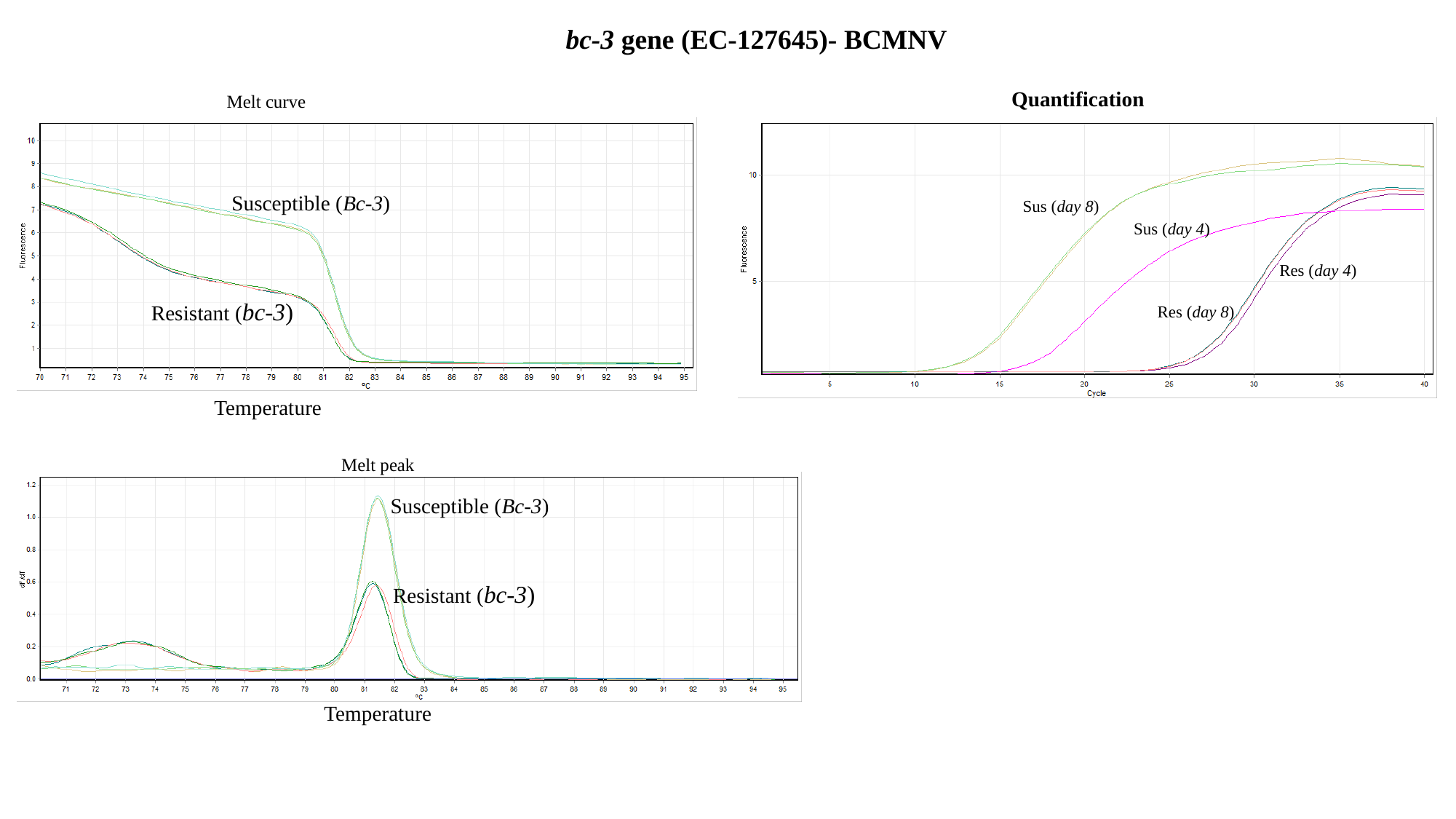

bc-3 gene (EC-127645)- BCMNV
Quantification
Melt curve
Susceptible (Bc-3)
Sus (day 8)
Sus (day 4)
Res (day 4)
Resistant (bc-3)
Res (day 8)
Temperature
Melt peak
Susceptible (Bc-3)
Resistant (bc-3)
Temperature

## Slide 11
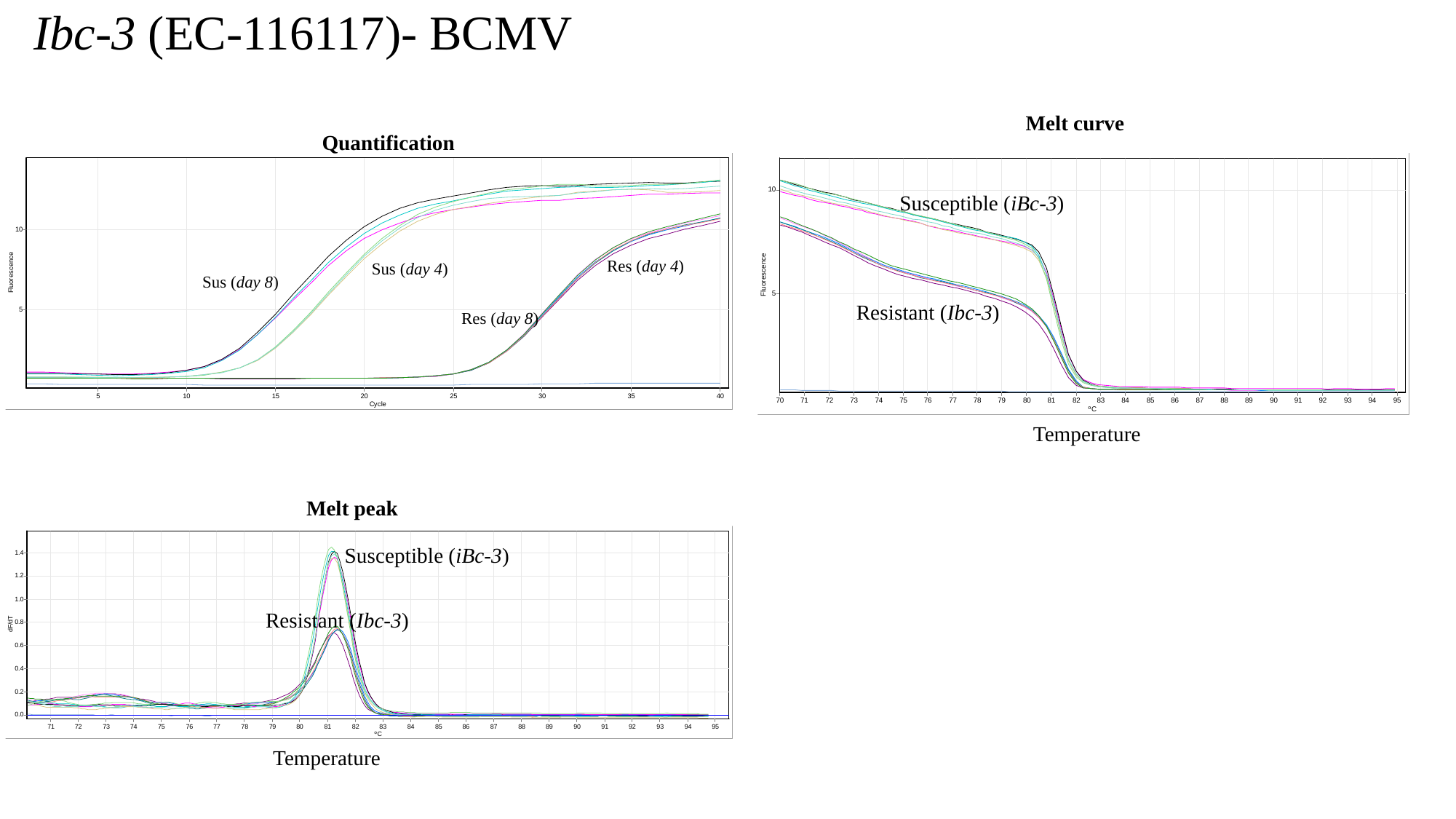

# Ibc-3 (EC-116117)- BCMV
Melt curve
Quantification
Susceptible (iBc-3)
Res (day 4)
Sus (day 4)
Sus (day 8)
Resistant (Ibc-3)
Res (day 8)
Temperature
Melt peak
Susceptible (iBc-3)
Resistant (Ibc-3)
Temperature

## Slide 12
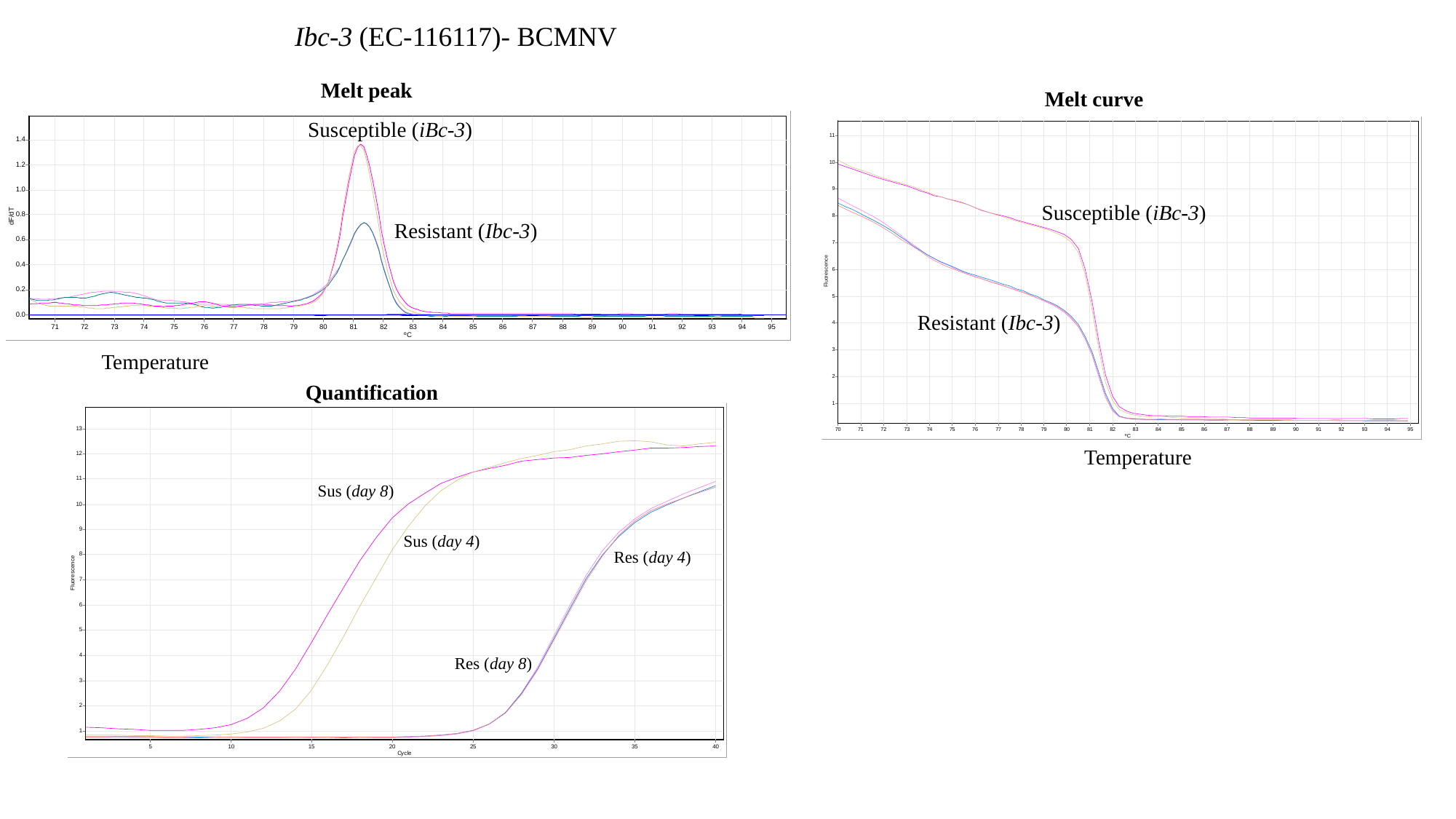

Ibc-3 (EC-116117)- BCMNV
Melt peak
Melt curve
Susceptible (iBc-3)
Susceptible (iBc-3)
Resistant (Ibc-3)
Resistant (Ibc-3)
Temperature
Quantification
Temperature
Sus (day 8)
Sus (day 4)
Res (day 4)
Res (day 8)
